# Vaptans and Hyponatremia in Critical Patients

**Published:** 2012-04-30

**Authors:** David D’Auria, Geremia Zito Marinosci, Giuseppe De Benedictis, Ornella Piazza

**Affiliations:** 1 Department of Anesthesia and Intensive Care, Federico II University, Napoli, Italy; 2 Department of Medicine, University of Salerno, Salerno, Italy

**Keywords:** AVP receptor antagonists, hyponatremia, vaptans, vasopressin

## Abstract

Hyponatremia is the most frequent fluid and electrolyte disorder in hospitalized patients (20%), particularly in ICU, associated with an increase in morbility and mortality. While hypovolaemic hyponatremia needs to be corrected with the replacement of the lost extracellular fluid by isotonic saline, euvolaemic (SIADH) and hypervolaemic hyponatremia (oedematous states like decompensated heart failure, liver cirrhosis, i.e.) are treated by restriction of fluid intake, loop diuretics and hypertonic saline. A novel approach consists in use of vaptans, non-peptide arginine vasopressin (AVP) receptor antagonists. Vaptans cause “aquaresis”, which results in the correction of plasma osmolality and serum sodium levels, without activation of the renin-angiotensin-aldosterone system or changes in blood pressure and renal function. In this paper we critically reviewed the results of the available randomized controlled critical trials, discussing the effectiveness and safety of vaptans in treating hypervolaemic and euvolaemic hyponatremia in critical patients.

## I. INTRODUCTION

### HYPONATREMIA: DEFINITION, SYMPTOMS AND CLASSIFICATIONS

Hyponatremia, defined as a serum sodium level below 135 mEq/l, is the most frequent electrolyte disorder and occurs in 3–15 % of hospitalized patients, especially in the departments of Intensive Care, Cardiology, Pulmonology, Internal Medicine, Surgery (1, 2).

It is defined as acute or chronic if its duration is shorter or longer than 48 hours respectively (3). Its clinical manifestation depend on many factors (time of duration, velocity of sodium decrease, sodium levels, patients age, comorbidities) (3) and can give few and aspecific signs and symptoms (fatigue, anorexia, nausea, behavioral alterations) when sodium level is higher than 125 mEq/l or the hyponatremia is chronic or it may cause more serious derangements when sodium level is below 120 mEq/l or hyponatremia is acute (cerebral edema, encephalopathy, seizures, coma) (3, 4). Nevertheless, hyponatremia is a predictor of poor outcomes in patients with heart failure. Patients with heart failure and hyponatremia often have inappropriately elevated plasma vasopressin levels and significantly poorer long-term prognosis than those with heart failure and normal serum sodium levels. (5)

With regard to the osmolality, except for a few cases of hyperosmolality hyponatremia (due to hyperglycemia or mannitol or other osmotic agents that cause a shift of water from the intracellular to the extra-cellular compartment), it is more frequently hypoosmolar (i.e. renal failure, liver cirrhosis, cardiac failure, states of hypovolemia, SIADH).

The commonest classification of hyponatremia refers to volaemic state: a) “hypovolaemic hyponatremia” due to extracellular fluid loss (vomiting, diarrhea, excessive sweating, use of diuretics, salt wasting syndromes, adrenal insufficiency); b) “euvolaemic hyponatremia” observed in primary polydipsia, hypothyroidism and in SIADH, a disorder determined by an excessive hypothalamic or ectopic secretion of vasopressin, which can be caused by a large number of pathologies (pituitary adenoma, encephalitis, other CNS disorders; cancer such as lung cancer, pancreatic carcinoma, thymoma; pulmonary inflammatory states; drugs as DDAVP, oxytocin, carbamazepine, tryciclics, cyclophosphamide, tioridazine) ; c) “hypervolaemic hyponatremia”, also called diluitional, observed in oedematous states that cause a fluid overload: acute or chronic congestive heart failure (CHF), liver cirrhosis, acute and chronic renal failure. (Table I). (6)

### HYPONATREMIA: CONVENTIONAL THERAPY

The conventional treatments of hyponatraemia differ according to the underlying disease.

Hypovolaemic hyponatremia simply requires the administration of lost fluids in the form of isotonic saline. As far as euvolaemic and hypervolaemic hyponatremia are concerned, the use of hypertonic saline solution is reserved for severe hyponatremia with neurological symptoms. The correction of the sodium intake should be slow and gradual and not exceed 8–12 mEq/l in the first 24 hours in case of acute hyponatremia, even slower in correcting chronic hyponatraemia (maximum 8 mEq/l in 24 h). Overly rapid correction, in fact, can cause serious and irreversible neurological disorders, such as the pontine myelinolysis due to brain glial damage (oligodendrocytes) (7, 8, 9, 10, 11). Osmotic demyelination syndrome results in confusion, behavioral changes, dysphagia, dysarthria, mutism, spastic hemiplegia or quadriplegia, convulsions, coma, death (3). Other conventional treatments used in managing hyponatremia consist in water restriction and loop diuretics (oedematous states). Lithium and Demeclocycline, capable of correcting hyponatremia causing nephrogenic insipidus diabetes, are rarely used because of their side effects and only under certain conditions (SIADH). Mannitol and urea (12) can be used to induce osmotic diuresis with increased excretion of free water.

Most of these therapies are of moderate efficacy. In any case it is important to monitor blood volume and assess frequently sodium levels (i.e. more a day by ABG), as well as other electrolytes, urine output, renal and cardiovascular function when correcting the serum sodium. None of the conventional therapies directly addresses the effects of elevated vasopressin levels, which is the underlying cause of many cases of hyponatremia.

### ARGININE VASOPRESSIN AND VAPTANS

Arginine Vasopressin or ADH (Antidiuretic hormone) is a cyclic peptide (9 amino-acids) with a disulfide bridge. It is synthesized in the hypothalamus (sopraoptic and paraventricular nuclei) and transported along the axons to the posterior lobe of hypophysis. It is released into the circulation in response to a number of stimuli (13). These stimuli can be osmotic (osmoreceptors situated in the anterior hypothalamus) or non-osmotic (low-pressure baroreceptors, present in large veins, right and left atrium, lungs; high-pressure receptors, present in the carotid sinuses and aortic arch). Other non-osmotic stimuli are hypovolemia, stress, hypoglycemia, nausea and vomiting, drugs. Circulating ADH is metabolized by a vasopressinase (also called oxytocinase or placental leucine aminopeptidase), widely expressed on cellular membrane. Well known ADH receptors are: V1a, V1b (also known as V3), V2. They belong to G-protein-coupled receptor superfamily. V1a and V1b receptors activate the phospholipase C/protein kinase C pathway, while V2 receptor activates the cAMP/protein kinase A pathway.

V1a receptor (expressed on surface of vascular smooth muscle cells, cardiomyocytes, platelets, hepatocytes, brain and muscle uterine cells) regulates vasoconstriction, inotropic effect, platelets aggregation, glycogenolysis, anxiety and stress, myocyte hypertrophy (14). V1b receptor, found in anterior pituitary gland, increases release of ACTH (15). V2 receptor is expressed on endothelial cells, where it determines release of von Willebrand factor, and on the basolateral membrane of the principal cells of the renal collecting ducts, where determines a water reabsorbition through the synthesis and insertion in the luminal membrane of the water channel aquaporina-2 (AQP2) (16).

According to antidiuretic action of V2 receptor, since the 80’s a large number of vasopressin antagonists has been investigated to treat hyponatremia. Because of limits linked to their peptide structure, the first peptide vasopressin antagonists were abandoned in favor of non-peptide vasopressin antagonists, also known as vaptans.

Non-peptidic vasopressin antagonists (i.e. vaptans) are: mozavaptan (OPC 31260) (17), lixivaptan (VPA 985) (18), conivaptan (YM 087) (19), satavaptan (SR 121463) (20), tolvaptan (OPC 41061) (21). (TABLE II).

Until now two vaptans have been released for clinic use: conivaptan, as an intravenous preparation, and tolvaptan, as an oral tablet.

Conivaptan (Vaprisol®) was the first medication of this class to be approved by the FDA in 2005 for the treatment of hyponatremia in SIADH, hypothyroidism, pulmonary disorders and adrenal insufficiency in hospitalized patients (22) and two years later, in 2007, also for hypervolemic hyponatremia in hospitalized patients (23). The loading dose is 20 mg i.v. over 30 min and the maintenance dose is 20–40 mg i.v. for continuous infusion over 24h up to 96h. Differently from all the other vaptans, which are selective V2-receptor antagonists, conivaptan acts like a V1a-receptor and V2-receptor antagonist. This singularity could be investigated in a randomized trial to show if conivaptan is better than other vaptans in treating hyponatremic patients with CHF thanks to its cardiovascular action (V1a-receptor blockage causes reduction of vascular peripheral resistance and inhibits unfavorable myocardial remodeling). It is metabolized by CYP3A4. Common side effects are infusion site reactions, fever, hypokalemia, hypotension, gastrointestinal disturbances. Udelson reported that a single iv dose of conivaptan increased urine output and reduced PCWP (pulmonary capillary wedge pressure) in patients with advanced heart failure (NYHA class III or IV) (46).

Tolvaptan (Samsca®) was approved by FDA for treatment of euvolemic and hypervolemic hyponatremia in HF, cirrhosis and SIADH, and by EMEA for treatment of SIADH (24, 25) only. The initial dosage is 15 mg once daily, increasing to 30–60 mg/day depending on clinical response. Like conivaptan it is metabolized largely by CYP3A4, so there are some warnings about interactions. The main adverse reactions are weakness, dry mouth, thirst, constipation, hyperglycemia.

## II. METHODOLOGY

### SEARCH STRATEGY

We searched CENTRAL (The Cochrane Central Register of Controlled Trials) and PubMed databases from January 2000 to January 2012. We searched for “vasopressin receptor antagonists” AND “hyponatremia”. In PubMed we applied filter for “Randomized Clinical Trial” and “Controlled Clinical Trial”. No language restrictions were imposed. According to these criteria 17 trials are described in this review.

In table III (Results) these studies, sorted in chronological order, are described and commented in a systematic manner, according to a scheme that shows references; the typology of study; the vaptan used, the population enrolled and size of the two groups (drug versus placebo); the pharmacological intervention and the primary and secondary outcomes (when provided); results; observations.

## III. RESULTS

**Table:** 

**a) Reference** **b) Typology of study**	**a) Vaptan** **b) Population** **c) Number of patients**	**a) Interventation** **b) Time** **c) Main Outcome**	**Results**	**Observations**
Gheorghiade M (Circulation 2003). (26) double-blind, multicentric, RCCT.	Tolvaptan Hyponatremia in chronic HF. n=254 patients: - n=191 tolvaptan (n=64: 30 mg n=64: 45 mg n=63: 60 mg). - n=63 placebo.	fixed doses of 30, 45, 60 mg/die without fluidic restriction and with stable furosemide doses. 25 days. Primary efficacy variable: change in body weight at day 1 vs baseline.	A decrease in body weight and an increase in urine output doses-related (p<0.001 for all treated vs placebo). Decrease in edema and normalization of s[Na^+^] in the tolvaptan group but not in the placebo group. Not significant changes in HR, AP, s[K^+^], renal function.	-A significant number of patients had only mild CHF and modest volume overload. -Necessity of further studies with NYHA-status stratification. -Focused only on diuretic properties of tolvaptan witout mentioning neurohormonal effects.
Gerbes AL (Gastroenterology 2003). (27) double-blind, multicentric, RCCT.	Lixivaptan. Hyponatremia in liver cirrhosis and ascites. n=60 patients: - n=40 lixivaptan (n=22: 100 mg n=18. 200mg). - n=20 placebo.	Primary end-point: s[Na^+^] ≥ 136 mmol/l. 100mg/die per os vs 200mg/die per os vs placebo with water restriction (max 1l/die). 7 days. s[Na^+^] normalization in 2 following measurements.	Normalization in 27% 100 mg/die group vs 50% in 200 mg/die vs 0% in placebo, respectively p<0.05 and 0.001.	-little study. -lixivaptan treatment was associated with a slight decline (8%) of the GFR.
Wong F (Hepatolgy 2003). (28) double-blind, multicentric, RCCT.	Lixivaptan. Hyponatremia in liver cirrhosis, HF, SIADH. n=44 patients: - n=33 lixivaptan (n=12: 25 mg n=11: 125 mg n=10: 250 mg). - n=11 placebo.	fixed doses of 25mg/bid vs 125 mg/bid vs 250 mg/bid per os vs placebo with water restriction (max 1.5 l/die) and diuretics. 7 days. changes in s[Na^+^], body weight, AP, diuresis.	Primary end-point: increase dose-dependent aquaresis (p<0.05).	-little study. -with higher doses appeared side effects that determined suspension of treatment in 5 patients.
Schrier RW (Study of Ascending Levels of Tolvaptan in Hyponatremia (SALT I+II); N Engl J Med 2006). (29) double-blind, multicentric, RCCT.	Tolvaptan. Hyponatremia in liver cirrhosis, HF, SIADH. n=448 patients: - n=225 tolvaptan - n=223 placebo.	increasing doses of tolvaptan 15, 30, 60 mg/die per os in 30 days. 37 days (30 days of terapy and 7 days observation after discontinuation). -Mean outcome: daily s[Na^+^] -Secondary Outcome: changes in body weight, total changes in s[Na^+^].	-Tolvaptan is effective in normalize s[Na^+^] vs placebo (134–135 vs 130 mEq/l at the 4^th^ day and 136 vs 131 mEq/l at 30^th^ day). (p<0.001 for all comparisons). -Dry mouth and thrist s(Na^+^) ≥146 mEq/l in the first day (1.6%).	- The trial was conducted without fluidic restriction. -After discontinuation, hyponatremia recurred. -The correction of hyponatremia in patients assuming tolvaptan was associated with a significant improvement of the mental health status measured by the SF-12 Questionnaire. This was the first study showing the benefits of a long-term treatment of hyponatremia with an oral vaptan.
Gheorghiade M (Am J Cardiol 2006). (30) prospective, multicenter, randomized, active-controlled, open-label trial.	Tolvaptan. Hyponatremia in liver cirrhosis, HF, SIADH. n=28 patients: - n=17 tolvaptan. - n=11 placebo + water restriction.	increasing doses of tolvaptan 15, 30, 45, 60 mg/die per os in 14 days. Follow up in out-patient clinic. 65 days. - Primary outcome: normalization of or a s[Na^+^] ≥ 10% increase from baseline. -Secondary outcome: changes in s[Na^+^], CH2O, plasma osmolarity and thrist.	Tolvaptan appears to be more effective than fluid restriction at correcting hyponatremia in hospitalized subjects, without an increase in adverse events. (p<0.05).	-little study
Soupart A (Clin J Am Soc Nephrol 2006). (31) double-blind (1st phase) and open-label (2nd phase), multicentric, RCCT. (phase II).	Satavaptan. Hyponatremia in SIADH. c.1) 1^st^ phase. n=34 patients: - n=26 satavaptan (n=14: 25 mg n=12: 50 mg). - n=8 placebo. c.2) 2^nd^ phase: n=22 patients.	1^st^ phase: fixed dose of satavaptan 25 or 50 mg/die per os. 2^nd^ phase: increasing doses of 12.5, 25, 50 mg/die with fluidic restriction (< 1.5 l/die). 1^st^ phase: 7–30 days 2^nd^ phase: 12 months s[Na^+^] normalization in first 24h, body weight, AP.	Significative increase in s(Na^+^) in patients assuming satavaptan 25 mg (p<0.01) and 50 mg (p<0.001) vs placebo. During the long-term treatment, 15 of 18 enrolled patients achieved 6 mo and 10 achieved 12 mo of treatment.	-The first trial showing a long-term effectiveness. -However 10% of treated patients reported an overly rapid correction of s[Na^+^] (> 12 mEq/l/day), no osmotic demyelisation syndrome was observed. -No drug-related serious adverse events were recorded during the long-term treatment and the s(Na^+^) response was maintained with a good tolerance.
Ghali J (J Clin Endocrinol Metab 2006). (32) double-blind, multicentric, RCCT.	Conivaptan Hyponatremia in liver cirrhosis, HF, SIADH. n=74 patients: - n=51conivaptan (n=24: 40 mg n=27: 80 mg). - n=23 placebo.	fixed doses of conivaptan 40 and 80 mg/die in two doses. Max water intake 2 l/die 5 days. change from baseline in s[Na^+^] area under the curve.	conivaptan shows a dose dependent increasing s[Na^+^] vs placebo (p<0.001). Headache, hypotension, nausea, constipation, and postural hypotension were the most common adverse events.	Thirst not included in side effects.
Konstam MA (The Efficacy of Vasopressin Antagonism in Heart Failure Outcome Study With Tolvaptan (EVEREST), JAMA 2007). (33) event-driven, double-blind, multicentric, RCCT.	Tolvaptan. Patients hospitalized for exacerbation of chronic HF with signs of volume overload, ejection fraction ≤40%, NYHA class III or IV simptoms. n=4133 patients: - n=2072 tolvaptan. - n=2061 placebo.	fixed doses tolvaptan 30 mg/die or placebo in addition to standard therapy. at least 60 days Outcomes c.1) Dual Primary outcomes: -1) all-cause mortality (superiority and noninferiority) -2) cardiovascular death or hospitalization for heart failure (superiority only). c.2) Secondary outcomes: - changes in dyspnea, body weight, and edema.	During a median follow-up of 9.9 months, 537 patients (25.9%) in the tolvaptan group and 543 (26.3%) in the placebo group died. The end points of cardiovascular mortality, cardiovascular death or hospitalization, and worsening heart failure were not different. Tolvaptan significantly improved secondary end points of day 1 patient-assessed dyspnea, day 1 body weight, and day 7 edema. In patients with hyponatremia, serum sodium levels significantly increased. Tolvaptan caused increased thirst and dry mouth, but frequencies of major adverse events were similar in the 2 groups.	In this trial tolvaptan was initiated for acute treatment of patients hospitalized with heart failure. The study, with a large number of patients and a long-term follow-up, shows that tolvaptan has no effect on long-term mortality or heart failure-related morbidity; but it also shows that tolvaptan is the first drug ever evaluated in patients hospitalized for worsening HF for which combination of short-term symptomatic benefits (↓ dyspnea, edema, body weight) and long-term safety has been established.
a) Zelster D (Am J Nephrol 2007). (34) c) double-blind, multicentric, RCCT.	Conivaptan. Hyponatremia in liver cirrhosis, HF, SIADH. n=84 patients: - n=55 conivaptan (n=29: 40 mg n=26: 80 mg). - n=29 placebo.	20 mg i.v. bolus followed by a continuos 96h i.v. infusion of 40 mg/die and 80 mg/die with fluidic restriction (max 2 l/die). 96 h Primary outcome: change in s[Na^+^], measured by the baseline-adjusted area under the s[Na^+^]-time curve. Secondary outcome: changes in s[Na^+^], osmolarity, ADH, vasoactives hormones.	Both conivaptan doses increased area under the [Na+]-time curve during the 4-day treatment (p < 0.0001 vs. placebo). Conivaptan significantly improved all secondary efficacy measures (p < 0.001 vs. placebo, both doses).	-This trial shows the rapid effectiveness of the conivaptan in the short-term treatment of hyponatremia. -Thirst’s increase is not considered.
Ginès P (HypoCAT; Hepatology 2008). (35) double-blind, multicentric, RCCT.	Satavaptan. Hyponatremia in liver cirrhosis with ascites. n=110 patients: - n=82 satavaptan (n=28: 5 mg/die n=26: 12.5 mg/d n=28: 25 mg/di) - n=28 placebo	fixed doses of satavaptan 5, 12.5, 25 mg/die of with fluidic restriction (max 1.5 l/die). All patients received spironolactone at 100 mg/day. 14 days. Main Outcome: changes in body weight from baseline (day 1) to the end of treatment (day 14) and chenges in s[Na^+^] from baseline to day 5.	Improved control of ascites, as indicated by a reduction in body weight (p = 0.05 for a dose-effect relationship overall) and improvements in serum sodium (p< 0.01 for all groups compared to placebo). Thirst significantly more common in patients treated with satavaptan compared to placebo. The frequency of other adverse events was similar among groups.	-The trial shows that satavaptan improves the control of ascites in cirrhotic patients under diuretic treatment and serum sodium dose-dependently. -Short-term study.
Annane D (Am J Med Sci 2009). (36) double-blind, multicentric, RCCT.	Conivaptan Hyponatremia in liver cirrhosis, HF, SIADH. n=83 patients: - n=53 conivaptan (n=27: 40 mg n=26: 80 mg). - n=30 placebo.	fixed doses of oral conivaptan 40 and 80 mg/die with oral fluidic restriction (max 2l/die). 5 days. Main outcome: baseline-adjusted area under the s[Na^+^]-time curve.	A normal s[Na^+^] or an increase from baseline ≥ 6 mEq/L was significantly higher among patients given conivaptan 40 and 80 mg/die (67% and 88%, respectively) than placebo (20%; P < 0.001).	- Short-term study. -Not evaluated the effects of other drugs allowed.
Wong F (J Hepatol 2010). (37) double-blind, multicentric, RCCT.	Satavaptan Ascites recurrence after paracentesis in cirrhotic patients. n=151 patients: - n=115 satavapta (n=39: 5 mg n=36: 12.5 mg n=40: 25 mg). - n=36 placebo	fixed doses of satavaptan 5, 12.5, 25 mg/die or placebo and spironolactone 100 mg/die. 12 weeks. frequency of paracentesis	The frequency of paracentesis was decreased significantly (n all satavaptan groups vs placebo (p<0.05). Increases in serum creatinine, orthostatic changes in systolic pressure and thirst were more common with satavaptan.	The trial included patients with or without hyponatraemia, and normal to mildly abnormal renal function.
Naidech AM (Neurocritic Care 2010). (38) prospective, randomized pilot (goal N = 20) trial	Conivaptan. neuro-ICU patients with severe hyponatremia (< 130 mE/l) or Hyponatremia (<135 mEq/l) with depressed GCS. n= 20	Conivaptan bolus (20 mg iv) followed by 20 mg IV over 24 h. 36h changes in serum and urine electrolytes and clinical examinations.	Conivaptan led to higher s[Na^+^] compared to usual care at 6, 24, 36 h (p<0.05).	Recruitment according to inclusion-criteria was difficult: the study was terminated after 6 patients were enrolled. Not conclusive results.
Abraham WT (The BALANCE Study: Treatment of Hyponatremia Based on Lixivaptan in NYHA Class III/IV Cardiac Patient Evaluation; Clin Transl Sci 2010). (39) double-blind, multicentric, RCCT. (phase III trial).	Lixivaptan. patients hospitalized for CHF (NYHA III-IV). n=652 patients: - n=326 lixivaptan - n=326 placebo	doses of lixivaptan or placebo adjusted on s[Na^+^] or volume status. 60 days. Increase in s[Na^+^] from baseline. Body weight and clinical measures.	Lixivaptan led to higher s[Na^+^] and reduce body weight, without renal dysfunction or hypokalemia.	BALANCE seeks to address unmet questions regarding the use of vasopressin antagonists including their effects on cognitive function
Aronson D (Short-and long-term treatment of dilutional hyponatraemia with satavaptan: the DILIPO study; Eur J Heart Fail 2011). (40) double-blind, multicentric, RCCT.	Satavaptan. diluitional hyponatremia (<135 mEq/l) in CHF. n= 118	Satavaptan 25 mg/die vs 50 mg/die vs placebo. 4 days (double-blind treatment), followed by non-comparative open-label satavaptan therapy for up to 343 days. s[Na^+^] ≥ 135 mEq/l and/or an increase in ≥ 5 mEq/l above baseline. Clinical measures.	The response rate was significantly (p<0.05) and doses-related higher with satavaptan than with placebo. Sodium responses maintained during open-label therapy. Higher rates of adverse events with the 50 mg/day dose.	The long-term open-label treatment results demonstrate sustained efficacy of satavaptan in maintaining normal sodium levels.
Galton C (Neurocrit Care 2011). (41) Open-label randomized controlled trial.	Conivaptan Within 24 h of severe traumatic brain injury. n=10 patients: - n=5: conivaptan - n=5: only usual care	a single dose 20 mg conivaptan. 48 h s[Na^+^], sodium load, change in ICP, urine output.	At 4 h, serum sodium was higher (P = 0.02) and ICP was lower (P = 0.046) in the conivaptan group. 24-h but not 48-h urine output was different between the two groups (P < 0.01 and P = 0.20, respectively). No drug-related serious adverse events	- non-hyponatremic patients enrolled. - little study. - further investigations are needed to assess the role of conivaptan in the management of intracranial hypertension.
Koren MJ (Am J Health Syst Pharm, 2011). (42) RCCT.	Conivaptan. Euvolemic or hypervolemic hyponatremia. n=49 patients.	Conivaptan 20 mg/die or 20 mg/bid or placebo via 30-minute i.v. infusion. 48 h. Change in s[Na^+^] from baseline to 48 hours.	Changes were significantly greater and dose-related compared with those in the placebo group. Both conivaptan regimens were more efficacious than placebo in all secondary efficacy outcomes. Conivaptan was generally well tolerated, with infusion-site reactions being the most common adverse effects.	

## IV. DISCUSSION

The analysis of the mentioned trials shows that non-peptide vasopressin antagonists are safe and effective drugs in the short-term correction of hyponatremia in both the states of hypervolemia (liver cirrhosis and heart failure) and euvolemia (SIADH).

Certainly vaptans have a number of limitations. First of all, trials presented a percentage of 10–20% of patients who do not respond to treatment. The reason for this phenomenon has not yet been studied well. This can be partly attributed to altered pharmacokinetics (altered volume of distribution in oedematous states and water ingestion), in part to polymorphisms and mutations in the vasopressin receptors (43, 44). On the other hand, vaptans may be associated with rapid correction of sodium values (> 12 h mEq/l/24) with potential risk of pontine myelinolysis, although the literature has not reported any cases of this complication in patients treated with these drugs.

Clinical response to vaptans in terms of aquaresis and hyponatremia correction is not the same in all subsets of patients. Patients with SIADH are those that respond best, while those with liver cirrhosis worse. Probably in the state of hypervolemia (CHF, liver cirrhosis) proximal tubular reabsorption reduces the effect that vaptans cause in distal tubular ducts. Further studies are needed to clarify this point. So clinicians need to keep in mind that therapy with vaptans should be individualized for each patient.

All studies were short term. Only Soupart (31) and Aronson (40) studied the effectiveness of satavaptan for one year, in SIADH and CHF respectively, demonstrating the efficacy and good tolerability in long-term treatment.

There are no studies comparing the efficacy between vaptans and traditional treatment (diuretics, hypertonic saline, urea,…) or between two different vaptans. This last point is considerable because, whereas conivaptan and mozavaptan (used in Japan for the treatment of hyponatremia in paraneoplastic SIADH)also block the V1a receptors, they may have superior efficacy compared to satavaptan, tolvaptan and lixivaptan (which are selective V2-receptor antagonists) in patients with heart failure.

All vaptans induce thirst and this effect is very important for compliance to therapy. Without doubt, the thirst is explained by the increased plasma osmolality that they determine, but it may be also linked to a direct action on vasopressin receptors of “thirst” in hypothalamic neurons.

In recent years the use of vaptans for treatment of emergency hyponatremia (45) and for the control of serum sodium and intracranial hypertension in neurosurgical patients has been discussed (38, 41). For the correction of emergency hyponatremia, hypertonic saline remains the treatment of choice, but it has been hypothesized that vaptans could at least be taken into account to give a slower correction of sodium rate than hypertonic saline and no risk of pulmonary edema (45). With regard to the treatment of cerebral edema and intracranial hypertension in neurosurgical patients, the current target is to keep the patient “salty and full”, with careful monitoring and rapid intervention to keep within normal limits the plasma osmolality and serum sodium (as well as the optimization of respiratory and cardiovascular functions, in addition to sedation, to guaranty an adequate DO_2_/VO_2_) through the consolidated use of mannitol, hypertonic saline, diuretics. To the best of our knowledge, there are still no conclusive studies on these two issues and we wait for results in the coming years.

## V. CONCLUSIONS

Vaptans are safe and effective drugs in the short-term treatment of hyponatremia due to hypervolaemic edematous states (liver cirrhosis, heart failure) and euvolaemic states (SIADH). They are contraindicated in hypovolaemic hyponatremia. About superiority of one vaptan above the other and comparison between vaptans and conventional therapy, questions are still open.

## Figures and Tables

**TABLE I:** CLASSIFICATION OF HYPONATREMIA ACCORDING TO VOLAEMIC STATE

**Volemic state**	**Hypovolemia**			**Euvolemia**			**Hypervolemia**
***Related Diseases***	***ECF loss, diarrhea, diuretics.***	***Salt wasting syndromes.***	***Adrenal insufficiency.***	***Hypotiroidism, primary polydipsia.***	***Drug, nausea, hypocortisolemia.***	***SIADH***	***CHF, liver cirrhosis, nephrotic syndrome.***
Edema	−	−	−	−	−	−	+
Blood pressure	Low	low	low	normal/low	Normal/low	normal	low
Urine sodium excretion	Low	high	high	low	high	high (low in type C)	low
Plasma renin activity	High	high	high	low	low	low	high
Others typical alterations:	hypercloremic metabolic acidosis in diarrhea; hypokaliemic metabolic alkalosis in diuretics.	response to saline infusion	hormone dosing, hyperkalemia	hormone dosing	hormone dosing	low blood urea and uricemia	

**TABLE II:** VAPTANS

	***Lixivaptan***	***Tolvaptan***	***Conivaptan***	***Satavaptan***
***V1a:V2 affinity***	1:100	1:29	10:1	1:112
***Route of administration***	oral	oral	intravenous	oral
***Dosage***	twice a day	once a day	continuous infusion	once a day
***Na^+^excretion/24 h***	↔ (small dose) ↑ (bigger dose)	↔	↔	↔
***Aquaresis effect***	+	+	+	+
***Administration***	SIADH, CHF, cirrhosis	SIADH, CHF, cirrhosis	SIADH, CHF, cirrhosis	SIADH
